# Two-year observation of the occlusal vertical dimension after bite raising via cone-beam computerized tomography: A preliminary study

**DOI:** 10.1038/s41598-019-39662-9

**Published:** 2019-03-05

**Authors:** Chuanzi Liu, Dan Huang, Lizhi Zhou, Guochen Liu, Yining Wang, Tao Jiang

**Affiliations:** 1 The State Key Laboratory Breeding Base of Basic Science of Stomatology (Hubei-MOST) & Key Laboratory of Oral Biomedicine Ministry of Education, School & Hospital of Stomatology, Wuhan University, Wuhan, China; 2 Department of Stomatology, Tongji Hospital, Tongji Medical College, Huazhong University of Science and Technology, Wuhan, China; 3 Department of Biostatistics, School of Public Health, Southern Medical University, Guangzhou, China; 4 Department of Prosthodontics, Hospital of Stomatology, Wuhan University, Wuhan, China

## Abstract

Variation of the occlusal vertical dimension (OVD) has been discussed empirically for decades, but it has not been thoroughly explored with experimental data. In the present study, cone-beam computerized tomography (CBCT) of six selected patients was conducted to evaluate the bones of the lower facial structures. The anterior lower facial heights (ALFHs), alveolar process heights (APHs), vertical facial pattern (VFP), occluding dentition height (ODH) and condyle space (CS), which were evaluated by three-dimensional (3D) and two-dimensional (2D) lateral cephalometry derived from CBCT, were compared before and two years after the OVD increased full mouth rehabilitation. Consistent significant increases in ALFHs and the VFP indicated the OVD increase, while a significant decrease in the ΔODH indicated compressed dentition. In addition, 55 of the 56 sites (98.21%) of APH measurement illustrated no significant difference before and after treatment. The findings indicated that the increased OVD did not relapse to baseline and was sufficiently tolerated, with mostly constant APHs and an altered ODH after two years of observation in the six patients.

## Introduction

The occlusal vertical dimension (OVD) is defined as the distance between two selected anatomical or marked points^1^. Although it has been widely accepted as one of the decisive considerations in oral rehabilitation, whether an increased OVD can be conserved continues to be debated^1–4^. Some investigators believe that because of alveolar bone remodeling, the OVD is stably sustained throughout life^4–9^. However, results from other studies have indicated that the height of the alveolar process remains constant^2,10,11^. As the OVD in the anterior lower face is mostly determined by the alveolar process heights (APHs) and occluding dentition height (ODH)^12^, with fixed APHs and a lengthening crown height, the tendency of the OVD to increase challenges the theory of a constant OVD.

In current clinical practice, the two abovementioned viewpoints introduced two major strategies, subtraction and addition^12–14^. Subtraction refers to reducing the opposite tooth to ensure adequate space without changing the OVD, while addition treatment refers to increasing the OVD to create extra space for a prosthesis to restore lost structures^12–14^. According to the description of the constant OVD, increasing the OVD is not only futile but sometimes dangerous^6,8^. Therefore, more endodontics and crown lengthening are suggested. Aesthetic demands, such as a deep bite of the anterior teeth and a wrinkled lower face, should not be considered^12,14^. However, the findings of other scholars have suggested that bite raising was predictable and safe. In Rebibo’s opinion, practitioners can manage the OVD under certain constrains^3^. With the extra space created by an increased OVD, endodontic damage from extensive oral rehabilitation can be reduced, and not only functional but also aesthetic restorations, which are considered an additional advantage of bite raising, can be achieved^13^. Thus far, no ideal treatment considering simultaneous damage prevention and structural preservation is available, and bite raising remains the best alternative strategy^15^. As different choices may lead to various outcomes, the implications for clinical work require further critical elucidation.

In analyzing the studies concerning the changing nature of the OVD, we find that most were indirect studies, such as skull evaluations^11,16^, animal studies^17^, and partial appliance studies^15,18,19^. To date, no conclusive evidence is available to support or refute the constant OVD theory or a reliable increase in the OVD, possibly due to difficulty in sample collection and bone marking problems. Benefiting from current cone-beam computerized tomography (CBCT) and three-dimensional (3D) cephalometry, detailed bone observation can be performed noninvasively^20–23^. The ability of 3D cephalometry to analyze bone structures such as the maxilla incisive canal and the mental foramen, and the benefit for bone surgical planning have already been shown^24–26^.

In the present study, analyses of cone-beam computerized tomography (CBCT) before and after bite raising were performed to study OVD changes in oral rehabilitation patients to provide evidence to resolve associated debates and develop guidelines for clinical practice.

## Results

**Figure 1:**
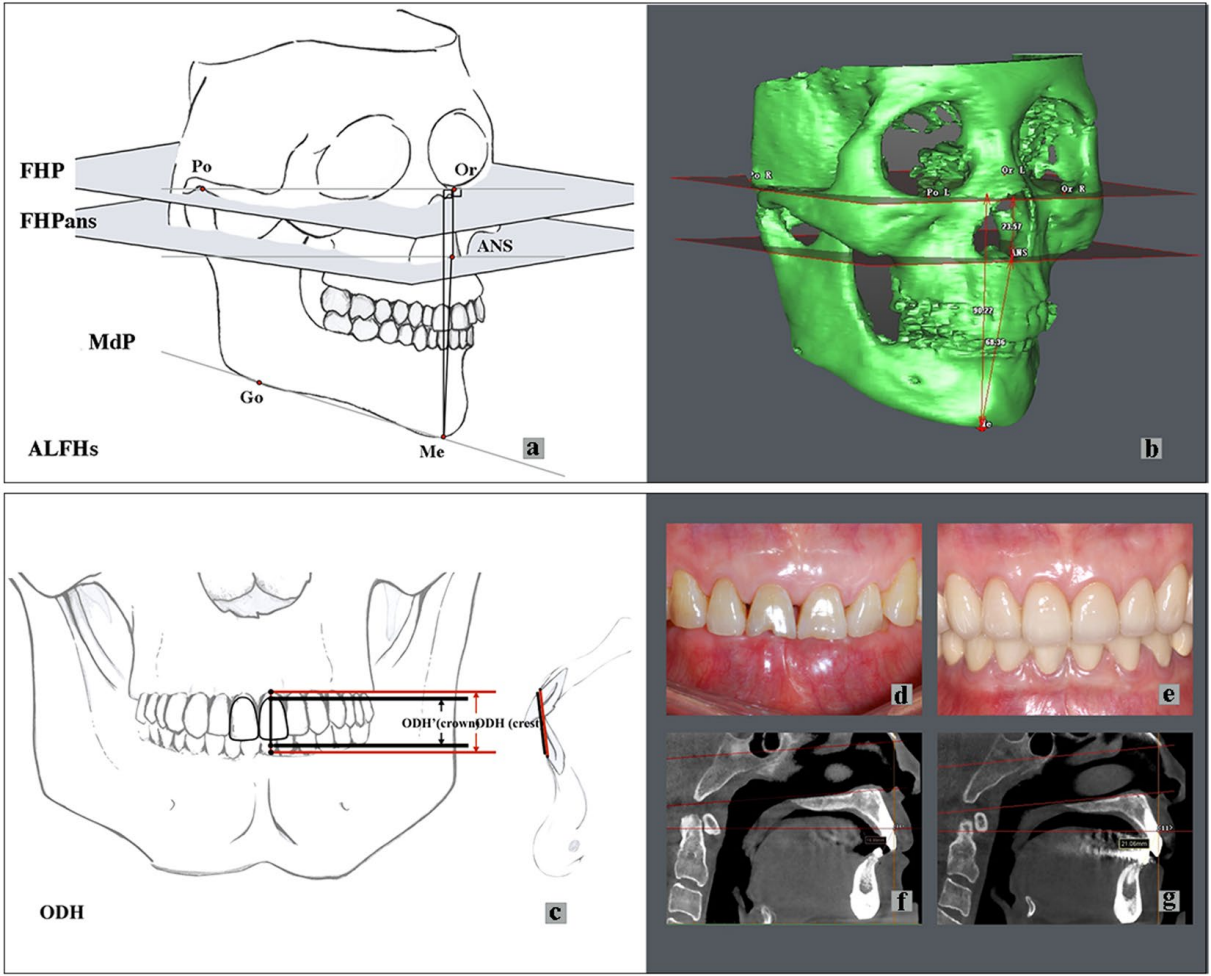
The measurements of ALPHs and ODH. (**a**,**b**) Measurements of anterior lower facial heights (ALFHs). See Table 1 for landmark definitions (FHP, FHPans, Po, Or, ANS, Me, Go, and MdP). (**c**) Measurements of occluding dentition height (ODH). The red lines “” represent the crest height and the black lines “” represents the clinical crown length. (**d**,**e**) Intraoral pictures before and after bite raising with respect to the ODH’ (crown); (**f**,**g**). 3D measurements of the ODH (crest).

**Figure 2:**
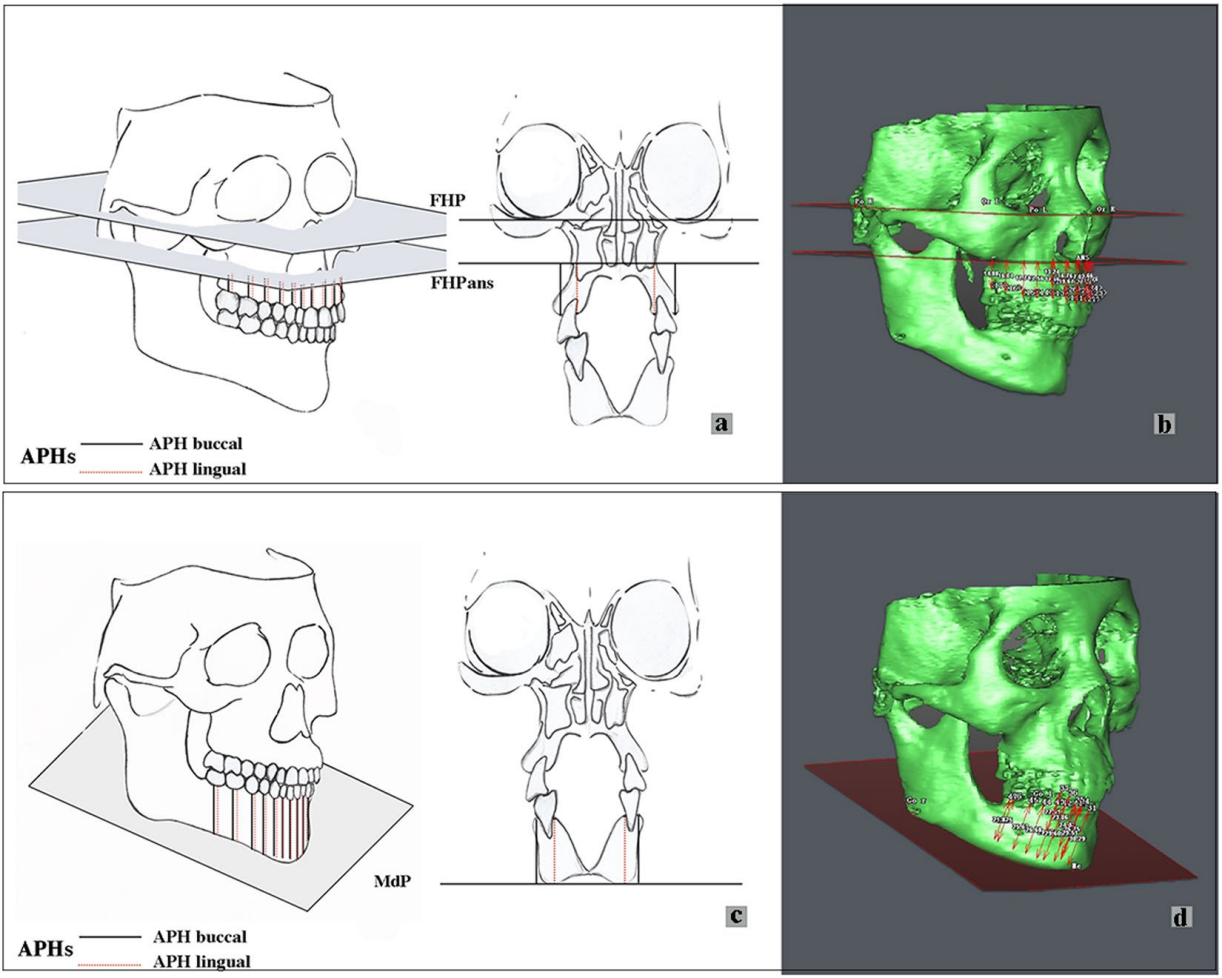
The measurements of APHs in the maxilla and mandible. (**a**,**b**). Measurements of alveolar process heights (APHs) in the maxilla; (**c**,**d**). Measurements of APHs in the mandible. The black lines “” represents the buccal heights and the red lines “” represent the lingual heights. See Table 1 for landmarks definitions (FHP, FHPans, MdP).

A total of six patients, including three males and three females with an average age of 51.8 years, were enrolled. The six patients had undergone fixed full-arch rehabilitation treatment for two years. After the application of provisional restorations, the ODH had been increased by 4.5–6.0 mm in the anterior teeth. Three-dimensional transformations of CBCT images to DICOM forms was performed before and two years after the raising procedure (Figs 1(b), 2(b,d)), and cephalometric landmarks were defined (Table 1).

**Table 1:** Definitions of the landmarks.

Abbreviations	Landmarks, reference planes and measurements	Definitions
Me	Menton	Most inferior point on the outline of the anterior bottom mandible.
Go	Gonion	Geometrically constructed as the point of intersection of the posterior and lower border tangents to the mandible.
ANS	Anterior nasal spine	The most anterior midpoint of the anterior nasal spine of the maxilla.
Po	Porion	Point on the upper margin of the external acoustic meatus, perpendicular to its center.
Or	Orbital	Lowest point of the floor of the orbit.
APHs	Alveolar process heights	The distance from the middle buccal and lingual points of a specific tooth to the alveolar bone base line.
FHP	Frankfort horizontal plane	The plane across the bilateral orbital and the left porion.
FHPans	Plane parallel to FHP and across the ANS point	Plane paralleled to the Frankford plane and across the anterior nasal spine point.
MdP	Mandibular plane	The plane across the bilateral gonions and the menton point.
FHP-GoMe	The angle of the FHP and GoMe plane	The angle of the Frankford plane and the GoMe plane.
Me-FHP	Me to Frankford plane	The vertical distance from menton point to the Frankford plane.
Me-ANS	Me to ANS	The linear distance from menton point to the anterior nasal spine point.
Me-ANS’	Vertical Me to ANS	The result of the Me-FHP subtracting ANS-FHP.
ANS-FHP	ANS to FHP	The vertical distance from the anterior nasal spine point to the Frankford plane.

### Anterior lower facial heights (ALFHs) and alveolar process heights (APHs)

The intra-observer reliability (ICC) was high for all 60 ALFHs and APHs, with an ICC ranging from 0.936 to 0.999 and a mean ICC of 0.993. The ICC of the four ALFHs ranged from 0.947 to 0.999, with an average ICC of 0.984, while the ICC of the 56 APHs sites ranged from 0.936 to 0.999, with a mean value of 0.994 (Table 2). Values near 0 (or negative) indicate poor reliability, and values close to one indicate high reliability.

**Table 2:** The ICC results of ALFHs and APH measurements.

Tooth	Buccal	Lingual	Tooth	Buccal	Lingual
11	0.936	0.999	21	0.997	0.998
12	0.999	0.996	22	0.993	0.998
13	0.999	0.999	23	0.999	0.999
14	0.990	0.994	24	0.985	0.975
15	0.997	0.998	25	0.996	0.997
16	0.997	0.999	26	0.998	0.999
17	0.997	0.998	27	0.998	0.998
31	0.998	0.985	41	0.998	0.998
32	0.997	0.946	42	0.997	0.965
33	0.995	0.994	43	0.997	0.997
34	0.993	0.998	44	0.998	0.997
35	0.991	0.999	45	0.996	0.998
36	0.992	0.998	46	0.998	0.998
37	0.997	0.999	47	0.996	0.995
Me-FHP	0.992	—	Me-ANS	0.999	—
ANS-FHP	0.947	—	Me-ANS’	0.999	—

Anterior lower facial heights (ALFHs) and other items (Me-ANS, Me-FHP, ANS-FHP, and Me-ANS’) are not present in the buccal or lingual region.

In paired t-tests of the ALFHs results, the *P* values were all below 0.05, indicating statistical significance (increases in the Me-ANS, Me-ANS’, and Me-FHP, and a decrease in the ANS-FHP). The results indicated the increase in the OVD (*P* < 0.05, Tables 1, 3). For the APHs, the results indicated that only one of the 56 measures (tooth position 35 in the lingual crest) was significantly different from the measurements before treatments at α = 0.05.

**Table 3:** The paired t-test results of ALFHs, APHs, ODH and VFP.

Measurement	N	Mean difference (mm (SD))	t-test (*P* < 0.05)	Mean difference (mm (SD))	t-test (*P* < 0.05)
		Buccal		Lingual	
11	5	0.25 (0.21)	0.06	0.66 (1.19)	0.28
12	5	0.12 (0.41)	0.54	0.34 (0.37)	0.11
13	6	0.81 (1.96)	0.36	1.45 (1.68)	0.09
14	6	0.27 (0.50)	0.25	−0.18 (1.56)	0.79
15	6	0.60 (0.78)	0.12	−0.02 (1.05)	0.96
16	5	−0.65 (1.28)	0.32	−0.52 (1.37)	0.44
17	4	−0.49 (1.12)	0.45	−0.28 (1.83)	0.78
21	6	0.08 (0.24)	0.47	−0.10 (0.55)	0.68
22	6	0.60 (0.58)	0.05	0.19 (0.58)	0.46
23	6	0.02 (1.41)	0.98	−0.03 (0.62)	0.91
24	6	0.02 (0.28)	0.99	0.08 (0.69)	0.80
25	6	−0.07 (0.56)	0.78	−0.03 (0.83)	0.93
26	5	−0.50 (1.01)	0.33	0.16 (0.42)	0.44
27	4	−0.36 (1.01)	0.53	−0.35 (0.51)	0.26
31	6	0.59 (1.23)	0.30	0.22 (0.96)	0.39
32	6	0.42 (1.22)	0.44	0.37 (0.95)	0.56
33	6	0.75 (0.91)	0.10	0.28 (1.07)	0.24
34	6	1.07 (1.25)	0.09	0.82 (1.51)	0.11
35	6	0.76 (0.99)	0.12	0.82 (1.03)	0.00*
36	6	0.59 (0.84)	0.15	1.06 (0.27)	0.40
37	6	0.37 (1.10)	0.45	0.41 (1.10)	0.27
41	6	2.29 (4.35)	0.25	0.12 (1.37)	0.84
42	6	0.60 (1.15)	0.26	−0.01 (1.30)	0.99
43	6	0.80 (1.35)	0.21	−0.03 (2.34)	0.98
44	6	0.32 (1.57)	0.64	0.16 (3.27)	0.91
45	6	0.35 (1.30)	0.54	0.74 (1.21)	0.19
46	6	0.77 (1.21)	0.18	0.64 (1.00)	0.18
47	6	0.09 (0.77)	0.79	−0.21 (1.12)	0.67
*Me-ANS*	6	−3.22 (0.73)	0.00*	—	—
*Me-FHP*	6	−2.61 (0.612)	0.00*	—	—
*ANS-FHP*	6	0.40 (0.35)	0.04*	—	—
*Me-ANS’*	6	−3.00 (0.71)	0.00*	—	—
*FHP-GoMe angles*	6	−2.33 (1.72)	0.02*	—	—
*ΔODH*	6	1.43 (0.85)	0.01*	—	—

A total of 56 sites (tooth positions 11–47) in the buccal and lingual regions were evaluated for alveolar process heights (APHs). Other items (Me-ANS, Me-FHP, ANS-FHP, Me-ANS’, ΔODH and FHP-GoMe angles) are not presented in the buccal or lingual region. *The difference was considered statistically significant at *P* < 0.05.

The 56 sites (tooth positions 11–47), both buccal and lingual, were evaluated for alveolar process heights (APHs). The Me-ANS, Me-FHP, ANS-FHP and Me-ANS’ in anterior lower facial heights (ALFHs) were evaluated on 3D models (Table 1). The ΔODH and FHP-GoMe angles were evaluated in the clinic and on 2D slices (Table 1). These six items were not present in the buccal or lingual regions.

### The occluding dentition height (ODH)

For the paired t-test results between the ∆ODH(T1-T0) and ∆ODH(T2-T0), the *P* value showed significant differences, suggesting that the ODH decreased after two years of observation (*P* < 0.05, Table 3).

### Vertical facial patterns (VFP)

From the data of the six cases, the FHP-GoMe angles significantly increased after the OVD increased (Table 1; *P* < 0.05, Table 3).

### Condyle space (CS)

Of the six patients, the anterior-posterior position in patients NOs. 1, 3 and 6 changed according to the linear percentage. In patient NO. 1, the left condyle moved posteriorly from the centric position. The bilateral anterior-located condyles in patient NO. 3 retreated to the centric positions, while the bilateral condyle positions both changed in patient NO. 6 (Table 4).

**Table 4:** The results of the condyle space (CS).

		T0 (before)				T2 (after 2 y)			
		AD (mm)	PD (mm)	LP (%)	Position	AD (mm)	PD (mm)	LP (%)	Position
**1**	**L**	2.94	2.35	11.15%	C	2.00	2.75	−15%	P
	**R**	2.35	2.06	6.58%	C	2.00	2.00	0.00%	C
**2**	**L**	2.86	3.10	−4.03%	C	2.75	2.63	2.23%	C
	**R**	3.64	4.09	−5.82%	C	2.41	3.01	−11.07%	C
**3**	**L**	2.27	1.33	26.11%	A	2.00	2.00	−4.76%	C
	**R**	3.20	1.22	44.80%	A	1.40	1.50	−3.45%	C
**4**	**L**	5.20	2.60	33.33%	A	5.29	2.94	28.55%	A
	**R**	6.40	2.00	52.38%	A	4.12	1.41	49.01%	A
**5**	**L**	1.88	2.19	−7.62%	C	2.00	1.60	11.11%	C
	**R**	1.88	1.87	0.27%	C	2.31	1.92	8.10%	C
**6**	**L**	2.45	2.07	8.41%	C	1.38	2.76	33.33%	A
	**R**	2.96	2.22	14.29%	A	2.86	2.86	0.00%	C

AD: anterior distance; PD: posterior distance; LP: linear percentage; L: left; R: right.

## Discussion

The existing debate regarding OVD remolding patterns has generated confusion in the oral rehabilitation field for decades^1–4^. However, few studies have focused on OVD changes after bite raising. Benefitting from CBCT and 3D cephalometric technology, for the first time, the present study noninvasively described the OVD bone landmarks. The OVD was not only shown by ALFHs at the macro level but was also analyzed in parts as a combination of APHs and ODH. The results revealed a solid OVD increase over two years in the six patients, with stable APHs and partial compression of the ODH.

In general, the most obvious finding was that the OVD represented by ALFHs significantly increased according to the Me-ANS, Me-FHP and Me-ANS’ data (Tables 1, 3). Moreover, the increased FHP-GoMe angles showed the OVD increase too (Tables 1, 3). Dahl B.L. *et al*., who studied the lateral cephalometric radiographs of twenty patients with anterior splints over sixty-seven months, also reported no relapse or rapid return to baseline^19^. Other scholars further demonstrated the adaptability of the masticatory muscles and neural system, implying that the OVD is variable and can be sustained within rational limits^1,27–29^. Empirical considerations regarding the fixed OVD may actually repudiate the variable nature of the dentoalveolar complex^30^.

Several methods are available to describe the lower facial height by marking soft tissue or bone structures. In clinical observations, OVD measurements on soft tissue may not be reliable, and as described by Gross *et al*., an increased OVD within 2 to 6 mm may not be visually distinguishable^31,32^. Therefore, in the present study, the linear distance (Me-ANS), vertical dimensions from points to planes (Me-FHP and Me-ANS) and cephalometric FHP-GoMe angles in bone structures were selected to represent the lower facial height. The consistent increases in these measurements provide convincing evidence proving that a sustained increase occurs.

In the analyses of parts, two major measurements, the APHs and ODH, should be considered^12^. Based on the anatomical structure, the anterior lower face height is separated into the APHs part and the ODH part^12^. Individually, APHs were unchanged in the paired t-test (Table 3). The results are consistent with some earlier tooth wear studies showing that the alveolar process heights remained constant throughout life^3,10,16,33–35^. A study of 244 medieval skulls showed that the distance between the IDC (inferior dental canal) and AC (alveolar crest) remained constant while the IDC to CEJ (cemento-enamel junction) increased with age, reflecting the eruption of teeth and the stable tendency of the alveolar process^10^. The compensation for OVD loss has also been suggested to reflect tooth movement without bone remolding^10,11^. Mostly, the masticatory muscles and neural system are sufficiently flexible to adapt to the newly changed OVD in the first several weeks^27–29^. However, although compensations occurs, it may not derive from variation in APHs. Considering the subject in this paper, the OVD was increased at the primary stage rather than decreased as the natural process of OVD loss. Whether the compensatory mechanism of OVD increasing is completely similar to that reported in previous researches remains uncertain. In this study, the results indicated that the APHs were constant and the tooth movements occurred in these six patients over two years of observation as discussed below.

To evaluate the tooth movement in bone, the ODH and ODH’, representing the upper crest occluding bone length and occluding clinical crown length, were measured. The significantly decreased ODH led to another interesting finding (Table 3). In the bone marked OVD, the teeth may have been compressed rather than the alveolar bones. Combined with the results of the CS, three of the six patients demonstrated anterior-posterior alterations (Table 4)^36^. In a monkey study by Ramfjord, the measurements in subject NO. 2 failed because of distortion of the mandiblular condyles^17^. Other scholars have also suggested the possibility of relative movement between the maxilla and mandible after bite raising^1,3^. In the present study, the relocated maxilla-mandible relationship may have introduced corresponding tooth movements such as tooth inclination and eruption. These tooth micro-adjustments are thought to be the primary factors in dental-alveolar adaptation^37^. The ultimate outcome of tooth movement may turn to be a compressed ODH.

In addition, the ANS-FPH, which was measured for the first time in this study, was found to be compressed as the OVD increased (Table 3). Usually, the ANS-FPH is measured as a connection between the lower and upper face. Separation of this part may provide a new perspective to observe the variations of the facial bones.

All these different aspects, as mentioned above, led to the conclusion that the increased OVD after bite raising was mostly maintained, with the constant APHs and an adjusted ODH in the six patients after two years. The results will provide a more profound understanding of bite raising in oral rehabilitation.

However, this study had limitations. A short time to reocclusion has been proven in several studies^38,39^, and a longer relapse time has been speculated to show no sign of reversion^17^. Two years of observations in this study may not be sufficiently long to draw a conclusion with certainty. In addition, the small sample size should also be cautiously considered when drawing conclusions. To further explore OVD changes, longer observation periods and a larger sample size should be considered.

## Conclusions

This preliminary study provides deeper insights into the study of OVD changes after bite raising. The methods of measuring parameters on CBCT images were found to be highly reliable. Overall, the results indicated that an increased OVD did not relapse to the baseline, and that the increased OVD was largely maintained. Additionally, the ODH was compressed partially and APHs remained constant over the two years of observation in the six patients, suggesting that the increase in the OVD was reliable during the observation period. This study also provides supports for the use of bite raising in oral rehabilitation, although further in-depth studies are needed.

## Method and Materials

### Ethics statement

This study was approved by the Ethics Committee of Stomatology Hospital, Wuhan University, China (NO. 2018 B08). The included patients all provided informed consent for their medical records containing the CBCT images to be used in scientific researches. The research methods were designed and performed in accordance with the STrengthening the Reporting of OBservational studies in Epidemiology (STROBE) checklist.

### Case selection

The medical histories of patients at the Prosthodontics Department of the Stomatology Hospital of Wuhan University over four years (from 2013 to 2017) were reviewed, and the CBCT images before and after treatment of dentate patients who had an increased OVD during oral rehabilitation were included. All included patients had been diagnosed according to the research diagnostic criteria for temporomandibular joint diseases (RDC/TMD) to detect temporomandibular joint (TMJ) conditions^40^.

The inclusion criteria were as follows:

1. patients without a history of temporomandibular joint diseases (TMD);
2. patients who had not undergone orthodontic surgeries in the maxillofacial bones or restorative procedures on more than two teeth in each quadrant;
3. CBCT was integrated in the zones of the TMJ and the lower face; and
4. patients who were classified as skeletal class I.

The exclusion criteria were as follows:

1. patients with substantial jaw deformity;
2. patients with bone metabolic disease; and
3. patients diagnosed with periodontitis.

### Imaging modalities

**Figure 3:**
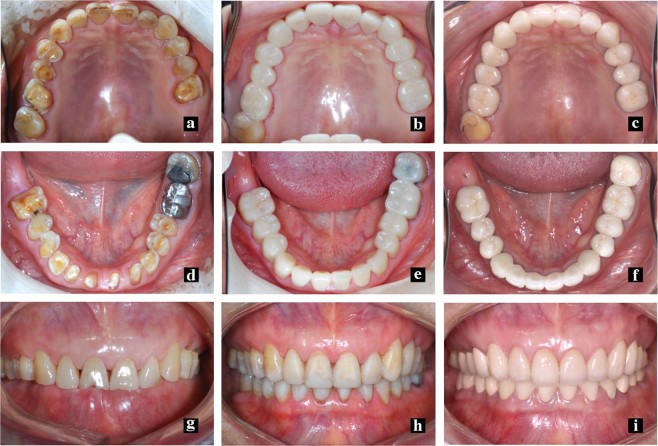
The clinical time points of treatments. (**a**,**d**,**g**) T0 (before treatments); (**b**,**e**,**h**) T1 (immediately after treatment) the resin provisional restorations were applied to increase the vertical dimension, and after 3–6 months, the fixed crowns were cemented; (**c**,**f**,**i**). T2 (the follow-up) two years after bite raising since T1.

**Figure 4:**
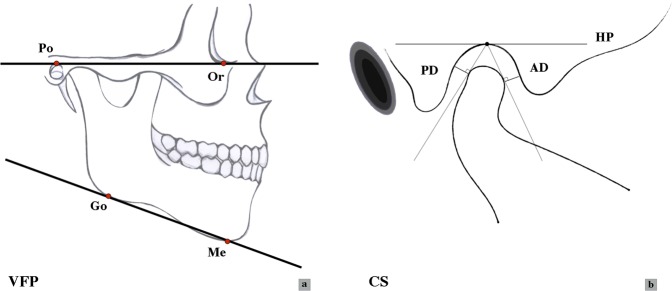
The measurements of VFP and CS. (**a**) Measurements of the vertical facial pattern (VFP). See Table 1 for landmarks definitions (Po, Or, Go, Me); (**b**) Measurements of the condyle space (CS) in the anterior and posterior directions. (AD: anterior distance, PD: posterior distance, HP: horizontal plane).

CBCT images were obtained at the Radiology Department, Stomatology Hospital, Wuhan University. The images were obtained using a high-resolution Newtom (QR s.r.l. Silvestrini, 2037135 Verona, Italy) with a 15 cm × 15 cm field of view, 110 kV, 2.6 mA and scanning time of 3.6 s. Both the axial pitch and thickness of each image were 0.30 mm. The CBCT images were then transformed into DICOM forms with a matrix setting of 512 × 512 pixels and were analyzed by SIM/Plant O&O (Materialize, Leuven, Belgium). Three time points were used in this study: T0 (before the treatment), T1 (immediately after treatment) and T2 (the follow-up) (Figs 1, 3). To observe the variations before (T0) and after the increase in OVD (T2), 3D reconstructions and 2D cephalometry derived from CBCT images were performed. The CBCT images immediately after the OVD increase (T1) were not taken because of the unnecessary radiation exposure. The ALFHs, APHs, VFP, ODH and CS were evaluated to explore multiple aspects of the OVD variations (Table 1; Figs 1, 2 and 4).

### Anterior lower facial heights (ALFHs) and alveolar process heights (APHs)

The plane parallel to the Frankfort plane (FHP) and crossing the ANS point (FHPans) was set as the bottom of the maxilla process, and the MdP was designed as the reference plane for the mandibular process height (Fig. 1a)^41–44^. In the present study, the ALFHs represented the bone marked OVD. To clearly describe the OVD, the Me-FHP, Me-ANS, Me-ANS’, and ANS-FHP were measured. The Me-ANS’ representing the vertical distance of ALFHs, was the distance from point Me to the ANS’ plane. The ANS-FHP represented the height of the nasal sinus part (Fig. 1(a,b)). For the APHs, the distances of all 28 tooth positions assigned by the FDI (Fédération Dentaire Internationale) numbering system were measured with the linear length from the middle buccal or middle lingual crest points to the corresponding alveolar planes (Fig. 2).

### The occluding dentition height (ODH)

To identify the change in the ODH after bite raising, the ODH(T0) (Fig. 1(d,f)), ODH(T1) and ODH(T2) (Fig. 1(e,g)) were analyzed as the distances between the upper and lower incisor crests in the CBCT images of the anterior teeth. According to the theory that the OVD reverts to baseline, no difference should exist between ODH(T0) and ODH(T2). If the ODH did not relapse or partially relapsed, then ∆ODH(T1-T0) and ∆ODH(T2-T0) should be compared. Because of the lack of CBCT images at T1, the ODH(T1) in this study was calculated by the ODH’(T1) (Fig. 3 (b,e,h)), which was represented by the clinical crowns in the anterior teeth at T1 (Fig. 1(d,e); 3) and determined from the medical histories of the subjects. As the patients with periodontitis had been excluded, recording the ODH(T1) as the ODH’(T1) plus twice the biological width and average gingiva depth^45^ (ODH(T1) = ODH’(T1) + 2*2.73 mm) was reasonable. The comparisons between ∆ODH(T1-T0) and ∆ODH(T2-T0) yielded relative values rather than absolute values. The defined differences in biological width and the average gingival depth were ignored in the comparison.

### Vertical facial pattern (VFP)

2D lateral cephalometry derived from 3D reconstructed images was calculated to acquire the VFP illustrated by the FHP-GoMe angle (Fig. 4a). The angles were classified into three categories: brachyfacial (<22°), mesofacial (22°–29°) and dolichofacial (>29°). The results indicated VFP changes before and after treatments^46^.

### Condyle space (CS)

As described by Pullinger and Hollender^36^, the condyle position was expressed as anterior, concentric, or posterior according to the following formula:

$${\rm{Linear}}\,{\rm{ratio}}\,({\rm{L}}{\rm{R}})=({\rm{P}}-{\rm{A}})/({\rm{P}}+{\rm{A}})\,\ast \,100 \% $$

where P is the linear posterior distance (PD) of the posterior line perpendicular to the tangent line from the point of contact (Fig. 4b) and A is the anterior distance (AD). According to the formula, when the linear ratio (LR) was lower than −12%, the condylar position was judged as posterior (P). When the LR was ±12%, the position was concentric (C), and if the ratio was higher than 12%, the condyle was anterior (A).

### Statistical methods

To verify the reliability of the methods used to obtain the ALFH and APH measurements, three patients’ CBCT images were measured by three operators. The ICC was evaluated for the three operators’ measurements. Two-way ANOVA without replication was applied to calculate the ICC for each item and site. Paired t-tests were performed to compare the mean differences in items before (T0) and after the OVD increase (T2) in the same patient. All statistical analyses were performed with SPSS 22.0 for Windows software (version 22, SPSS, Chicago, IL). Statistical significance was set at a two-tailed *P* value of 0.05.

## Supplementary information

STROBE_checklist_cohort.

SREP-18-23258C Author List Changes Approval form.

Original approval document.

Ethic approval English translation.

## Data Availability

All data generated or analyzed during this study are included in this published article.
