# Supplementary material for: Two-year observation of the occlusal vertical dimension after bite raising via cone-beam computerized tomography: A preliminary study
Source: Sci Rep. 2019 Mar 5;9:3509. doi: 10.1038/s41598-019-39662-9 (PMC6401134; doi:10.1038/s41598-019-39662-9)
Supplement: Supplementary file 2 — SREP-18-23258C Author List Changes Approval form. [file 41598_2019_39662_MOESM2_ESM.docx]

In accordance to Nature Publishing Groups Authorship Policy we agree to change the authors of the manuscript as indicated below.

**NAME OF JOURNAL**: Scientific Reports

**TITLE OF MANUSCRIPT**: Two-year observation of the occlusal vertical dimension after bite raising via cone-beam computerized tomography: a preliminary study

**MANUSCRIPT NUMBER**: **SREP-18-23258C**

**CORRESPONDING AUTHORS NAME: Tao Jiang**

**PREVIOUS AUTHOR NAMES:**

Chuanzi Liu, Dan Huang, Lizhi Zhou, Guochen Liu, Yining Wang, Tao Jiang

**UPDATED AUTHOR NAMES:**

Chuanzi Liu, Dan Huang, Lizhi Zhou, Guochen Liu, Yining Wang, Tao Jiang

**CHANGE TO AUTHOR LIST:** The institution of Dan Huang changes from “The State Key Laboratory Breeding Base of Basic Science of Stomatology (Hubei -MOST) & Key Laboratory of Oral Biomedicine Ministry of Education, School & Hospital of Stomatology, Wuhan University” to “Department of Stomatology, Tongji Hospital, Tongji Medical College, Huazhong University of Science and Technology, Wuhan, China”

| **Print Name** | **Signature** | **Date** |
| --- | --- | --- |
| Chuanzi Liu | 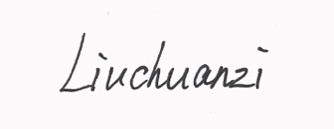 | 2018.10.16. |
| Dan Huang | 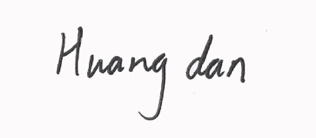 | 2018.10.16. |
| Lizhi Zhou | 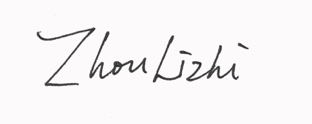 | 2018.10.16. |
| Guochen Liu | 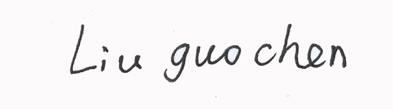 | 2018.10.16. |
| Yining Wang | 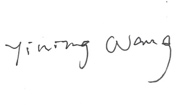 | 2018.10.16. |
| Tao Jiang | 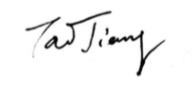 | 2018.10.16. |
