# Supplementary material for: Two-year observation of the occlusal vertical dimension after bite raising via cone-beam computerized tomography: A preliminary study
Source: Sci Rep. 2019 Mar 5;9:3509. doi: 10.1038/s41598-019-39662-9 (PMC6401134; doi:10.1038/s41598-019-39662-9)
Supplement: Supplementary file 3 — Original approval document. [file 41598_2019_39662_MOESM3_ESM.pdf]

武汉大学口腔医院医学伦理委员会审批件

[2018]伦审字（B08）号

项目名称	垂直距离升高咬合重建患者锥形束断层扫描的观察研究
试验目的	1□ 2□ 3□ 4□ 5□ 6□ 7□ 8□ 9□ 10□ 11☑
申请单位	武汉大学口腔医院
项目负责人	刘川子

内容提要

在咬合重建治疗中，是否升高垂直距离（oral vertical dimension OVD）长期存在着争议。一些学者认为，由于牙槽骨的代偿能力，升高的 OVD 能在术后自然恢复至原有的高度。然而，另一些学者发现，牙槽骨的高度存在终身不变的现象。OVD 主要由牙槽骨高度（Alveolar process heights APHs）及咬合牙列高度（Occluding dentition height ODH）决定，在 ODH 因临床修复体而升高，APHs 不变的情况下，OVD 必然会出现升高。在此理论下，OVD 不变的假说存在着不合理性。升高 OVD 与否，患者在临床上有着截然不同的结局。不升高 OVD，咬合重建患者为了开辟修复空间，将有可能选择大范围的根管治疗以及对合牙体的磨除。毫无疑问，这一选择有较大的创伤性。反之，合理升高 OVD，牙体组织的医源性创伤将最大化降低，在功能修复的同时，还可以兼顾美学修复。迄今为止，关于 OVD 升高后变化的研究数量仍较少，且大部分研究是间接进行的，比如动物实验，解剖学研究，部分牙列的升高等等。由于计算机辅助技术的发展，锥形束断层扫描（Cone beam computerized tomography CBCT）技术的成熟，使得 OVD 变化的研究可以通过 CBCT 无创性的进行。

本实验将搜集 OVD 升高咬合重建患者的术前及随访 CBCT，结合临床病历信息，通过对比来观察来探索 OVD 的变化规律，为咬合重建的临床治疗方案提供科学的理论基础。

申办者：刘川子

2018年 3 月 12日

伦理委员会审查意见

同意。

武汉大学口腔医院医学伦理委员会

（印章：武汉大学口腔医院国家药物临床试验机构 医学伦理委员会）

主任委员签名：[illegible]

2018年 3 月 27日

备注： 1=Ⅰ期临床试验； 2=Ⅱ期临床试验； 3=Ⅲ期临床试验； 4=Ⅳ期临床试验； 5=临床药代动力学试验； 6=已有国家标准的药品的临床试验； 7=进口药临床试验； 8=生物等效性； 9=医疗器械临床试验；10=国际多中心临床试验；11=其它;A 动物实验；B 人体样本。
