# Supplementary material for: Two-year observation of the occlusal vertical dimension after bite raising via cone-beam computerized tomography: A preliminary study
Source: Sci Rep. 2019 Mar 5;9:3509. doi: 10.1038/s41598-019-39662-9 (PMC6401134; doi:10.1038/s41598-019-39662-9)
Supplement: Supplementary file 4 — Ethic approval English translation. [file 41598_2019_39662_MOESM4_ESM.pdf]

THE HOSPITAL OF STOMATOLOGY, WUHAN UNIVERSITY

Application Form for Ethics Approval

NO. [2018] （ B08 ）

**Project name**	The observation of the bite raising patients via CBCT
**Objective**	1□ 2□ 3□ 4□ 5□ 6□ 7□ 8□ 9□ 10□ 11□✓
**Organization**	The hospital of stomatology, Wuhan University
**Project leader**	Chuanzi Liu

Objectives, research plan and methodology:

In the field of oral rehabilitation, there is a controversial debate about whether to increase the oral vertical dimension (OVD) or not. Some researchers believed that owning to the alveolar reconstruction, the raised OVD would relapsed to the basis. Notwithstanding, other researchers found that the heights of alveolar were constant through entire life. As illustrated above, the nature of OVD variation needs to be revealed.

Generally, the OVD is composed of alveolar process heights (APHs) and occluding dentition height (ODHs). As the increasing heights of restorations in dentition and the constant APHs, the theory of unchanging OVD is opposing challenge. To increase the OVD or not leads to significant different clinical consequents. Analyzing the studies implying the OVD changing nature, most of them are performed in indirect ways. Benefited from the three dimensional cephalometry nowadays, the bone observation can be applied in details and noninvasively.

In present study, analyses on CBCTs before and after bite raising had been performed to study the OVD changing in advanced TSL patients in order to provide evidence to explain the debates and guidelines for the clinical practice.

Applicant：Chuanzi Liu 2018/3/12

Investigation opinions of the Ethics Committee:
Approved
THE HOSPITAL OF STOMATOLOGY, WUHAN UNIVERSITY
ETHETIC COMMITTEE

Signature of the chairman: Wenfeng Zhang 2018/3/27

Remarks: 1=phase I clinical trials; 2=phase II clinical trials; 3=phase III clinical trials； 4=phase Ⅳ clinical

trials； 5=clinical pharmacokinetic studies； 6=national standard clinical pharmacokinetic studies; 7=clinical studies of imported drugs; 8=bioequivalence; 9=clinical studies for medical apparatus and instruments； 10=international multi centered clinical trials；11=others; A animal experiments；B human body samples.
